# Evaluation of CyberKnife Radiosurgery for Recurrent Trigeminal Neuralgia

**DOI:** 10.7759/cureus.2598

**Published:** 2018-05-09

**Authors:** Aldo Berti, George Ibars, Xiaodong Wu, Alex Sabo, Michelle Granville, Gail Suarez, James G Schwade, Robert E Jacobson

**Affiliations:** 1 Department Neurosurgery, University of Miami Hospital, Miami, USA; 2 Neurosurgery, South Miami Hospital, Cyberknife Center of Miami; 3 Innovative Cancer Institute, Innovative Cancer Institute, Cyberknife Center of Miami; 4 Neurology, Pain Management, Nova Southeast/larkin Community Hospital; 5 Miami Neurosurgical Center, University of Miami Hospital, Miami, USA; 6 Cyberknife Center, Miami, FIU; 7 Cyberknife Center of Miami, University of Miami Miller School of Medicine

**Keywords:** stereotactic radiosurgery, trigeminal neuralgia, recurrent trigeminal neuralgia, gammaknife, cyberknife

## Abstract

Stereotactic radiosurgery (SRS) has evolved as an accepted treatment for medication resistant trigeminal neuralgia. Initial results are very good but follow-up over three to five years shows a gradual return of pain in up to 50% of treated patients, often requiring further treatment. The results with repeat SRS using the isocentric Gamma Knife (GK) (Elekta, Stockholm, Sweden), especially in patients having initially good results, are very similar to the outcomes after the initial treatment although there is an increased risk of residual facial numbness secondary to the additional radiation dose to the trigeminal nerve. However, after 2000, non-isocentric SRS systems began to be used for treating trigeminal neuralgia including the CyberKnife (CK) (Accuray, Sunnyvale, California) as well as various linear accelerator (LINAC) based systems. This report specifically examines a series of recurrent trigeminal cases treated by the same group of physicians with the CK system. Similar doses and locations on the trigeminal nerve and/or the root entry zone were used for both initial and repeat SRS treatment regardless of system used. Although there are numerous series reporting the use of GK for recurrent treatment for recurrent trigeminal neuralgia, there are no series reviewing the results and long-term effectiveness using CK for repeat SRS for recurrent trigeminal pain. We reviewed 23 cases that had initial treatment for trigeminal neuralgia either surgically or with SRS with either the GK or CK and then a later second procedure only with CK. The follow-up after the second CK SRS ranged from three to 13 years found that the results are very similar to the multiple reports in the literature describing second or third SRS treatments with the GK. Results of repeat radiosurgery treatment of recurrent trigeminal neuralgia appear to be independent of the system used and are primarily based on proper target and dose to the trigeminal nerve.

## Introduction and background

Background of stereotactic radiation treatment of trigeminal neuralgia 

Stereotactic radiosurgery (SRS) has evolved as one of the main treatments for trigeminal neuralgia (TN) in combination with medical management. For almost 20 years, Gamma Knife, an isocentric Cobalt radiation unit (GK) (Elekta, Stockholm Sweden) was the initial radiosurgery method available for providing a highly focused isocentric radiation dose to a small target limited to four to six millimeters (mm) along the trigeminal nerve, labeled as stereotactic radiosurgery (SRS) [1-3]. The initial SRS experience reported, including follow-up results, has been extensively published and upgraded for over 30 years, and the range of initial treatment radiation dosages, targets, and results with one to 10 year follow-ups as well as the secondary effects, especially residual facial numbness, are well established [2,4,5]. The initial treatment dose is found to be an important factor, varying from 70 Gy to 90 Gy with a tendency to better relief but associated with a higher rate of numbness with the 90 Gy dose [1,4]. The accepted target is four to six mm along the trigeminal nerve, although some centers include one to two mm of the trigeminal root entry zone in the pons [6,7]. Some centers have found that higher initial dose to the trigeminal nerve and possibly early treatment also lead to better results [8,9]. The most common measurement scale to rate and access pain in trigeminal neuralgia is the Barrow Neurologic Institute (BNI) scale. This scale stages the patient from 0 to V using a combination of degree of residual pain and use of medication both before and after treatment. Excellent and good results are considered in the range of BNI 0-III indicating no pain or mild or intermittent pain controlled with medication. Post procedure follow-up results afterwards are similarly evaluated on the same scale [10].

Follow-up of treatment results in large series for initial treatment of trigeminal neuralgia using GK, at one year, show excellent to good results (BNI 0-II or even III a) between 72% to 86% of cases. Most large series have a small group with immediate pain relief within 48 hours of treatment but the majority of patients get results over the initial 30 days. Longer-term follow-up extending over three, five, and 10 years after GK SRS indicate that the results gradually deteriorate, resulting in residual excellent and good results (BNI 0-III) in only 44 to 50% five years after treatment [1,2,4]. There is a relationship between better long-term pain relief and development of mild post-procedure facial numbness, which is frequently related to extending the radiosurgical lesion one or two millimeters into the root entry zone [5,7,11-14].

In the past 15 years, as extensive experience developed with GK, and as the use of radiosurgery for treatment of trigeminal neuralgia was becoming common, experience has also evolved with using alternative radiosurgery systems such as the non-isocentric robotic arm CyberKnife (CK) (Accuray, Sunnyvale, California) that were reported were equivalent in range to the results achieved using GK [15-20]. A recent report of 138 cases using the CyberKnife for initial treatment of trigeminal neuralgia with a mean dose of 75 Gy had an effective pain control rate (BNI class I-IIIa) at six months of 93.5%, 12 months 85.8%, 24 months 79.7%, and at 36 months 76% [15]. As both clinical experience and the planning software and computer movement controls of these CK or LINAC systems have improved, the results for initial treatment of trigeminal neuralgia using equivalent dosing and targets are almost similar to the much larger reported GK experience [21-23]. As treatment teams, including the neurosurgeon, radiation oncologist, and radiation physicist, became more familiar with the treatment target and dose for trigeminal neuralgia, there are reports using different non-isocentric LINAC systems for the initial treatment of trigeminal neuralgia that match that achieved with both GK and CK [22-27]. In previous studies using stereotactic radiosurgery for cerebral lesions and comparing dosing there has been no difference between GK and CK [28,29]. As different non-isocentric and isocentric stereotactic radio surgical systems have become available, the decision to initially treat trigeminal neuralgia with GK, CK or even LINAC based systems may be a result of machine proximity, insurance coverage or physician training on the various systems rather than because there is any significant difference in results. Dosimetric comparisons have been made allowing conversion of dosing parameters based on the isodose lines from one system to another [30].

The use of SRS for repeat treatment for recurrent trigeminal neuralgia

Trigeminal neuralgia is an often recurrent disease regardless of success of initial treatments with medications such as carbamezapine and gabapentin, SRS, radiofrequency rhizotomy, balloon compression, or microvascular decompression. In follow-up after initial SRS, the majority of patients continue with good relief up to the second or third year and then the results gradually deteriorate with pain recurrence [31]. There are many treatment options for recurrent pain after the initial treatment, whether it is SRS, radiofrequency trigeminal rhizotomy, balloon compression, or microvascular decompression [32,33]. Many patients, even though they do not have severe episodic pain, often need supplemental medication including or gabapentin after treatment [11]. This persistence or development of low grade discomfort in the trigeminal area has been speculated to be related to possible gradual remyelination of the trigeminal nerve after radiation damage [12,33]. When pain recurs after three or more years, many GK patients undergo repeat SRS treatment, and numerous series using GK show that the outcome of a second SRS treatment is similar to the initial treatment with 61% excellent or good results within the initial 24 months but with a higher incidence of residual facial numbness [34-42]. There are even case reports of third SRS treatments within the dose restraints of multiple treatments with a debate of the proper dose relative to risk of secondary effects on the adjacent brainstem and the trigeminal nerve and risk of increased facial numbness [42,43]. Although there are numerous reports of series of patients treated with recurrent pain with GK, there are no series reporting the use of CK for recurrent trigeminal neuralgia after initial successful SRS treatment, which is the focus of this report.

## Review

There were 23 patients reviewed who underwent CK stereotactic radiosurgery for recurrent trigeminal neuralgia after previous radiosurgery. The cases came from a retrospective chart review at a single CyberKnife^R^ radiosurgery treatment center, referred by multiple neurosurgeons and neurologists over 10 years. The center was established in 2003 as an open staffing model. None of the physicians had any financial interest in the CK center except the director, a radiation oncologist, who was not a treating physician on any of the cases and whose role is explained in the financial disclosure section (JGS). All neurosurgeons had previous experience with GK radiosurgery. All radiation oncologists and physicists had worked with both GK as well as various types of radiation therapy units for cranial radiotherapy. Chart and patient review was made with patient consent when contacted as well as the consent of the treating neurosurgeon. The previous history and length of symptoms, medication used, BNI score if available, and previous treatments were tabulated including the original SRS dose and type of machine used. The interval from primary treatment to second treatment with CK was tabulated. The second treatment dose, total dose, and any side effects or symptoms were tabulated; the length of follow-up including any pain recurrence was determined directly through chart review or by phone contact (Table 1).

**Table 1:** Trigeminal neuralgia treatment table Table showing age of patient when CyberKnife treatment was given for recurrent trigmenal pain. Time (years) between treatments, dosage for each treatment, and total dose of treatment (CyberKnife/Gamma Knife) and post treatment pain free interval. The last four patients received other treatments such as radiofrequency ablation or microvascular decompression as the initial treatment prior to undergoing CyberKnife SRS. *Gamma Knife treatment TIME BT TX: Time between treatments. RF: Radiofrequency ablation to the trigeminal nerve. SX: Surgery (microvascular decompression).

SEX	AGE	1ST DOSE	TIME BT TX	2ND DOSE	TOTAL DOSE
M	80	75	3	65	140
M	75	71.67*	5, 3	65	136.67
F	72	80*	6	70	150
M	82	75	1	65	140
M	79	75*	9	65	140
F	85	80*	6	62	142
F	79	75*	7	70	145
F	69	75	2	70	145
F	68	80*	2	60	140
F	71	70	4	65	135
F	53	75*	6	65	140
F	78	70	4	76	146
M	51	70	< 1	60	130
F	73	75	1	60	135
F	84	75	4	60	135
M	70	80*	3	60	140
F	86	75	2	65	140
M	92	80*	11	69.5	149.5
F	79	80*,40*	10	30	150
F	77	3 RF	2	60	60
F	64	RF & SX	3,1	76	76
F	65	SX	5	75	75
F	87	SX	18	75	75

**Figure 1:**
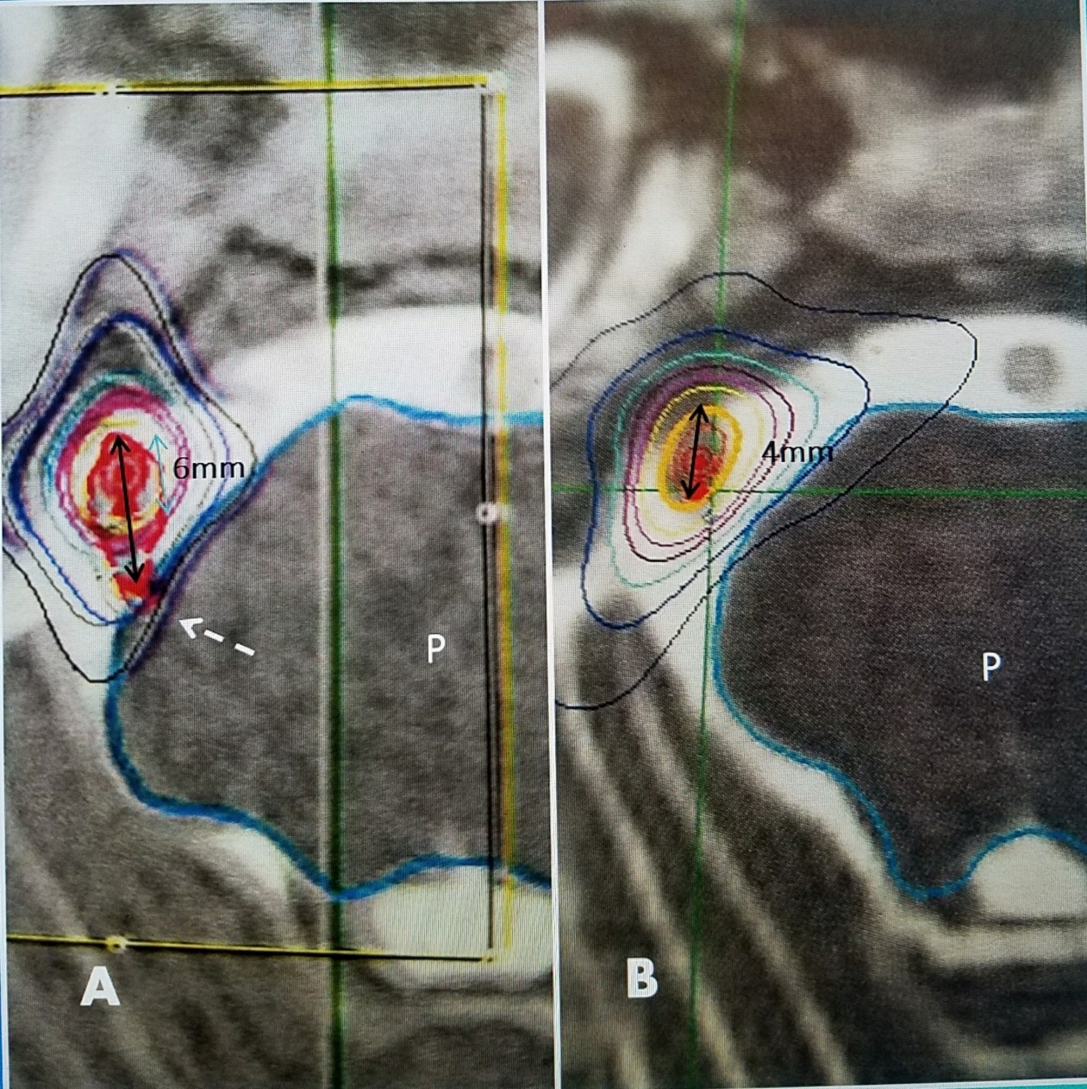
Initial and repeat stereotactic radiosurgery plans A: 2009: Initial CyberKnife treatment plan with 75 Gy at 100% line with 6 mm length of trigeminal nerve (solid black arrow) including 1 mm at the trigeminal root entry zone (dotted white arrow) at the lateral aspect of the pons (P). B: 2014: Repeat CyberKnife plan 5 years later with 65 Gy to 4 mm of the more distal trigeminal nerve (solid black arrow). The treatment field is oriented along the more distal length of the nerve.

The average age at the second treatment was 66.8 years. There were 17 females and six males. Nineteen of the 23 had either Gamma Knife or CyberKnife radiosurgery as the initial treatment for trigeminal pain. Of the 19 radiosurgery patients, seven had their initial treatment with the GK and 12 with the CyberKnife. One of the patients had the Gamma Knife treatment twice within six months; therefore, undergoing three total SRS treatments. The other four patients had either radiofrequency trigeminal rhizotomy or microvascular decompression as their initial treatment. All patients reviewed for this report had the second treatment for recurrent trigeminal neuralgia using the CyberKnife. The average interval of all patients until the second retreatment was 4.54 years. The interval before a second treatment after Gamma Knife SRS was 4.7 years and CyberKnife 4.45 years. Two radiosurgery patients had recurrent pain bilaterally at separate time intervals and had separate treatments for each side. The average initial SRS treatment dose was 70.9 Gy (ranging between 70 to 80 Gy). There was no dose difference in the initial SRS treatments between GK and CK. The average second dose with the CyberKnife was 63.76 Gy, 10% lower than the initial dose, ranging between 60 to 75 Gy. Review of the actual radiosurgery plans revealed that 18 of the 23 cases treated between four and six millimeters of the trigeminal nerve and only five of the 23 had any extension of the radiation field into the root entry zone of the adjacent pons (Figure 1).

The outcome and length of symptomatic pain relief after the second CyberKnife stereotactic radiosurgery was tracked in follow-up interviews and chart review. Six patients of the original 23 were lost to long-term follow-up because of age and extended time interval from repeat SRS to time of this review. However, chart information showed that they were pain free at least from two to three years after the repeat radiosurgery. The average trackable pain free interval of all repeat CK patients was 3.7 years. The shortest interval before a third treatment either with radiosurgery or radiofrequency trigeminal rhizotomy was six months. The longest follow-up pain free period was 12 years. Interestingly, all patients remained on baseline medication including either carbamazepine and/or gabapentin after the repeat SRS procedure. Although some patients were known to be clearly pain free after the second SRS, this retrospective review could not reveal if patients had episodic pain. As a result, since they remained on medication, they were classified as BNI III to IIIa after the second SRS with CK. Five patients of the 17, or 29%, that were trackable over two to 12 years had further repeat procedures for recurrent pain. Residual facial numbness was seen in four patients or 24% of the treated group. Three patients had later radiofrequency trigeminal rhizotomy rather than a third SRS treatment at three, four, and six years after the repeat procedure, one had a balloon compression and two patients were able to maintain pain control with peripheral trigeminal branch blocks. The small number of patients undergoing repeat CK SRS as a second treatment combined with a significant number lost to follow-up makes it impossible to perform statistical evaluation. However, the majority of patients where follow-up was available had at least a two to three year pain free interval. Over 12 years only 29% eventually had other procedures, which is consistent with what has been reported after repeat GK studies [1,5,33-40].

Discussion

In this follow-up group, there appears to be no short or long term difference in patient response for repeat SRS treatment using the CyberKnife compared to the published reports with the Gamma Knife. This small retrospective review indicates that the precision of the target and dose are the determining parameters of pain relief for recurrent trigeminal neuralgia rather than the particular radiation unit used. Detailed pre-treatment radiographic studies and mapping of the trigeminal nerve complex are critical in defining the precise target of the trigeminal nerve within the basal cisterns especially when using contrast enhancement and 3.0 Tesla imaging [44]. Treatment of recurrent pain after successful SRS with the GK has been extensively reported in the literature and a PubMed search found over 450 cases of repeat SRS with the GK but there are no specific reports of recurrent stereotactic radiotherapy using CK or LINAC based systems except for anecdotal mention within articles on treating trigeminal neuralgia [19-21]. The return of pain within the first year after SRS treatment has been classified either as immediate or short term failure.

In all large series, after initial success between 75 to 93%, the failure rate gradually increases with continued follow-up over three, five, and 10 years after the initial treatment, so that up to 50% of the patients with initial good results based on the BNI scale are faced with the need for possible retreatment [1,3-5]. Retreatment options and results after acute and short term failure are different than retreatment after sustained pain relief for many years [8,11]. Although repeat stereotactic treatment is used, patients with short-term failure often require radiofrequency rhizotomy or microvascular decompression [33-40]. Experience with GK has identified the issues affecting treatment and retreatment including the initial pain response, time interval after initial treatment for recurrence, the target, the dose/nerve volume treated and whether the radiosurgery target was the trigeminal nerve alone, the length of nerve treated and if the target was combined with 1-2 mm of the dorsal root entry zone, and the strength of the initial dose [6-8]. These same parameters apply to the treatment with second dose as much as the initial dose [12,33].

Development of numbness in the face is often listed as a complication but is more a secondary effect and many studies find that numbness after treatment correlates positively with good to excellent pain response [12,13,44]. The dose exposure of the adjacent brainstem is a critical factor in repeat SRS and related to post procedure facial numbness [45]. The total cumulative dose to the brainstem and cumulative doses to adjacent structures such as the temporal lobe must be carefully calculated [46,47]. Normally, the initial SRS dose with either GK or SRS varies between 70 to 90 Gy with most centers using a dose of 75 Gy [1,5]. However, the reported retreatment dose using the GK from large centers reporting hundreds of cases of trigeminal neuralgia varies from a low of 45 Gy to a high of 90 Gy combined with a determination to extend the target to the root entry zone in the second treatment if not done initially. Treating a longer segment of the trigeminal nerve may also allow lower dosing to be equally effective with less risk of facial numbness since radiation effect is dose and volume related [46]. The higher dose, both for initial and repeat treatment, is associated with a higher incidence of residual facial numbness varying from 12 to 30% [1,5,13,14]. There are reports of even third SRS treatments and a more individualized plan, especially targeting sections of the trigeminal nerve not in the original radiation plan in cases with recurrent pain [42,47,48]. Experience with treating multiple brain metastatic disease clearly demonstrates the feasibility of multiple repeat SRS treatments [49]. In repeat trigeminal SRS, the reported maximum combined dose limit appears to be 180 Gy with many centers favoring equivalent or higher dosing for the second treatment as long as the patient is aware of the increased risk of facial numbness, which parallels good pain relief [13,14,20]. It is possible with careful evaluation of the dosing to the adjacent brainstem, temporal lobe, cornea, and other critical structures that additional treatment beyond the second SRS can be tolerated with recurrent trigeminal neuralgia [41,42,48].

## Conclusions

The original experience using repeat stereotactic radiosurgery to treat recurrent trigeminal neuralgia was reported using the isocentric Gamma Knife and this series reviewed patients treated using the non-isocentric CyberKnife but using the same dose range and targets on the trigeminal nerve and the adjacent root entry zone. The results demonstrate similar effectiveness with excellent and good results in the immediate two to three years after the second SRS being about 60%. The longest pain free interval in follow-up after the repeat treatment was 12 years. The percentage of persistent facial numbness after the second procedure was also similar to that reported with GK. Interestingly, in this group, despite successful treatment with repeat radiosurgery, all patients were kept on at least a baseline dosage of carbamazepine or gabapentin to prevent pain recurrence. Although the use of medication classifies the patients after treatment as a BNI III or IIIa, the treating neurosurgeons felt it was beneficial to reduce the long-term risk of further pain recurrence. In this small group, only 26% of patients followed up to 15 years had further procedures after the second^ ^radiosurgery treatment, which may be related to the continued use of medication after the repeat SRS. In this series, the second^ ^treatment used an overall slightly lower average target dose than the initial stereotactic treatment. However, an extensive review of more recent literature regarding repeat SRS definitely supports the use of potentially higher doses for retreatment. Reports in the literature also indicate it is possible to even perform a third SRS treatment, as long as the patient is aware of an increased risk of residual or new facial numbness, the potential risk of developing malignant tumors in surrounding tissues, such as the temporal lobe. The dose spread to the brainstem and temporal lobe must be kept within accepted tolerance limits. This long-term follow-up series of a small group of patients receiving a second SRS treatment with CK after a previous SRS with either GK or CK demonstrates that CK is equally effective as the previous reports of repeat SRS using GK. Most importantly, it is the target and dose to the trigeminal nerve, with or without including the root entry zone, that is the key factor in obtaining effective pain relief in patients with recurrent trigeminal neuralgia rather than the particular radiosurgical system used.
